# Variations in healthcare utilization for mental health problems prior to suicide by socioeconomic status: a Norwegian register-based population study

**DOI:** 10.1186/s12913-024-11113-w

**Published:** 2024-05-21

**Authors:** Carine Øien-Ødegaard, Solveig Tobie Glestad Christiansen, Lars Johan Hauge, Kim Stene-Larsen, Sissel Marguerite Bélanger, Espen Bjertness, Anne Reneflot

**Affiliations:** 1 Division of Mental and Physical Health, Norwegian Institute of Public Health, PO Box 222, Skøyen, Oslo, 0213 Norway; 2 Department of Community Medicine and Global Health, University of Oslo, Institute of Health and Society, PO Box 1130, Blindern, Oslo, 0318 Norway; 3 Institute of Health and Society, Faculty of Medicine, University of Oslo, Oslo, Norway

**Keywords:** Suicide, Primary healthcare utilization, Mental healthcare utilization, SES, Educational attainment, Employment status, Income level

## Abstract

**Background:**

Suicide poses a major public health challenge, claiming around 650 lives annually in Norway. There is limited understanding of mental healthcare utilization patterns preceding suicide, particularly relating to socioeconomic status (SES). This study analyzes mental health service use among Norwegian citizens aged 20–64 from 2009 to 2021, emphasizing disparities related to SES.

**Methods:**

This is a population-wide registry-based study. We include mental health consultations with both primary and specialist healthcare services, and investigate patterns of service use regarding educational attainment, employment status and income and compare this to the population in general. All suicides in the period (*N* = 4731) are included in the study. The aim is to investigate potential discrepancies in service use the year and month preceding suicide, seeking to enhance targeted preventive interventions.

**Results:**

Our results show significant variations in healthcare use for mental health problems the last year preceding suicide, according to the components of SES, for both men and women. Those with higher education utilize the mental healthcare services prior to suicide to a higher degree than men and women with high school education or less, whereas employed men and men with high income level have significantly lower mental healthcare usage prior to suicide both the last year and month compared to the non-employed men and men with low-income level. Employed women also had a lower mental healthcare usage, whereas the results regarding income are not significant for women.

**Conclusion:**

Mental healthcare use prior to suicide varies across the SES components. Notably, the SES groups exhibit heterogeneity, with gendered patterns. Targeted interventions for low consultation rates among employed men, and men with high income and lower education are needed, while women, and men in at-risk groups, such as the non-employed and those with low income, demonstrate higher mental healthcare utilization, warranting comprehensive suicide prevention measures.

**Supplementary Information:**

The online version contains supplementary material available at 10.1186/s12913-024-11113-w.

## Background

Suicide is a major public health issue and continues to rank as one of the leading causes of lost years of life worldwide [1, 2]. It is a complex phenomenon that requires a variety of prevention measures, and despite both action plans and interventions, continues to be the cause of death for about 650 persons each year in Norway [3]. Several studies emphasize the importance of access to healthcare and improved quality of care as important strategies for suicide prevention [4–6]. This includes rightly identifying and supporting people in distress, but a prerequisite is that those in need utilize the healthcare services. It is common to be in contact with healthcare services prior to suicide. Up to four out of five are in contact with primary healthcare (PHC) services [5, 7–11], and about one out of three are in contact with specialized mental healthcare (MHC) services, during the last year prior to the suicide [4, 8]. There has been reported a more extensive healthcare use among women than among men, as well as an increasing consultation rate with age. However, there is a knowledge gap regarding variations in PHC and MHC consultations by socioeconomic status (SES).

Previous studies indicate high healthcare usage for mental health problems among those who die by suicide but a more detailed investigation of variations by SES is still lacking. Some studies suggest minimal SES-differences in general PHC usage [12, 13], but it has not been established whether this is applicable to the healthcare use preceding suicide. In some countries, such as Norway, PHC services are the most readily accessible healthcare services, and the general practitioner (GP) functions as a gatekeeper into specialist healthcare (SHC) services. Several studies report that low SES is associated with less contact with the SHC system, including MHC services [12–18], but it has not been determined if this also relates to MHC usage prior to suicide. There is a well-established relationship between low educational attainment, non-employment and/or low income and increased risk of suicide [19–23]. If individuals with low SES both utilize the mental healthcare system to a lesser extent and have an increased risk of suicide, there might be a missed potential for suicide prevention targeted towards this group of individuals, and thus, investigating inequalities in healthcare usage for mental health problems prior to suicide is vital.

Norway has a universal healthcare system, that aims to be accessible for all inhabitants, regardless of level of income [18]. However, data from several OECD-countries suggest that variations in SHC usage according to SES is common even in countries with universal healthcare [13]. Facilitators to care that have been identified in the literature are mental health literacy, a positive view of services and social support for help-seeking. These factors, however, are also highly related to determinants of SES, like educational attainment, employment status and income [24, 25].

Regarding PHC usage prior to suicide more specifically, this is generally high in Norway, at 80% and 89% the last year prior to suicide for men and women, respectively [8]. A review of MHC use among suicidal individuals found that about one third of those with past-year suicide ideation, plans and/or attempts were in contact with MHC the last year prior to suicide [26]. Regarding the association between SES and help seeking prior to suicide, previous research have found that suicide victims in the lowest quintile of SES have an increased odds of not contacting medical care [27].

Although some studies point to differences in healthcare use according to SES in general, a detailed investigation of between-group differences in healthcare use one year and one month prior to suicide is missing. Identifying SES groups who to a lesser extent utilize mental healthcare services is essential to implement targeted suicide prevention measures more effectively. Few studies, if any, on this topic have used population-wide registers, with the possibility to examine mental health consultations with both primary and specialist healthcare services prior to suicide. In the present study of all Norwegian citizens aged 20 to 64 years in the period 2009 to 2021, we aim at investigating patterns of healthcare use for mental health problems by determinants of SES (educational attainment, employment status and income) in the year and month preceding suicide. Additionally, we aim at comparing the use of healthcare for mental health problems one year prior to suicide with the usage of the same services in the general population in a calendar year.

## Population and methods

### Study design and registries

The design of the current study is longitudinal, and the results stem from data from several Norwegian administrative registries, which we have linked using deidentified unique identification numbers. We utilize data from the Cause of Death Registry (CDR), the Norwegian Patient Registry (NPR) for MHC consultations, the Control and Payment of Health Reimbursements Database (KUHR) for PHC consultations, the Norwegian Population Registry (DSF), Statistics Norway’s Educational Registration System, and FD-trygd (Social security database) from the Norwegian Labour and Welfare Administration (NAV). The dataset has one observation per person per year in the period 2009 to 2021, but in the case of death or emigration they are censored out the following year. The maximum observation length is 13 years, for both those who die by suicide and the population in general. All Norwegian residents aged 20 to 64, born between 1957 and 1991 are included. The dataset includes 2,732,621 persons, of which 4,731 die by suicide. There are 31,077,414 observations in total.

We used Chi-square tests for comparison of patterns of service use between the groups of suicide deaths and the population in general. To account for potential covariation and confounding effects, sensitivity analyses were done using logistic regression models, comparing PHC usage to no usage, MHC usage to no usage, and MHC usage to PHC usage. The covariates included are death by suicide, educational attainment, employment status, income level and age group. As the association between higher education and healthcare utilization for mental health problems was different for those who die by suicide and the general population, we included an interaction term in the models. The results are stratified by gender because of the known gender-differences in both suicide risk and healthcare use for mental health problems prior to suicide.

We have used Stata 17.0 to construct the record linkages and the analyses.

Ethical approval was obtained from the Regional Committee for Medical and Health Research Ethics, South East Norway (REK 2014/1970) and all registry owners approved utilization of their data. The study is part of a project funded by the Research Council of Norway (Treatpath, project number 288,731).

### Variables

Death by suicide is identified in the CDR as deaths classified by the International Classification of Diseases (ICD-10) codes X60–X84 and Y870. Since we focus on healthcare usage in relation to mental health problems, we only include PHC consultations with mental health diagnoses or symptoms, called a “P-diagnosis” from the International Classification of Primary Care (ICPC-2). In Norway, all medical contacts require a diagnostic code to be valid for reimbursement from the state, and because of this almost all consultations with PHC are registered with at least one diagnostic code. The PHC consultations can be with either a primary care physician, most often the GP, or emergency primary care. Data on MHC consultations are derived from NPR and cover children and adolescents’ psychiatric services (BUP), adult mental healthcare (VOP), and specialized substance use treatment (TSB). Due to suicide being a marginal phenomenon and low levels of MHC usage in the population in general, we have combined inpatient and outpatient consultations in the analyses. However, we also have performed analyses with two groups of MHC consultations, which can be found in the Supplementary material.

We have categorized healthcare usage for mental health problems into three groups. The first are those with at least one PHC consultation for mental health problems, but no MHC consultations. The second are those with at least one MHC consultation, regardless of additional PHC consultations. The last group are those without any consultations for mental health problems. We display the proportion within each of these categories at two timepoints for those who die by suicide. The first includes those with consultation(s) one to 365 days, and the second group one to 30 days prior to suicide. Since the population in general does not have a natural “end date”, we have calculated all the mental health consultations made by the general population within a calendar year. The results displayed for both the suicide deaths and the population in general are averaged across the observation period.

SES is a multifaceted concept, and we include three of the most common measures, educational attainment, employment status and total income. Educational level is binary, with the levels “High school or less” and “Higher education”. Higher education includes everyone with at least a bachelor’s degree. Information regarding educational level is limited to the period 2009 to 2020, so for 2021 we have used the highest completed level as of 2020.

Employment status is also a binary variable with the categories “No employment” and “Employment”. To be categorized as the latter the individual must work minimum one hour per week, which is also used as the definition of employment by Statistics Norway [28]. The majority of the employed have fulltime employment, and less than 5% of the employed works less than four hours per week. Information regarding employment is lagged one year. This variable is measured in October, so in the case of suicide prior to October the variable is missing in the year of death. Hence, we use data from the previous year.

Finally, we include total income for the individual. This variable includes both taxable income and welfare benefits. Total income is divided into three groups, indicating which income tertile in the population the individual belongs to. Income is also lagged one year, for the same reason as employment status. However, we argue that employment status and income level in the year prior to death contribute to a decent understanding of SES-differences in healthcare use for mental health problems prior to suicide.

Information regarding gender and age are found in the DSF. The information we have is legal gender, which in Norway consists of the two categories “man” and “woman”. Since 2016 the legal gender can easily be changed by the individual solely based on their own assessment of their gender identity. As suicide is a rare incident, we have included as many as possible from the adult population and have a wide age-span, from 20 to 64. Individuals younger than 20 are primarily in high school, while those older than 64 are primarily retired.

## Results

Table 1 below displays the distribution of the dataset across all the variables.

**Table 1:** Distribution of mental health consultations with MHC, PHC, educational attainment, employment status and income level, for those who die by suicide and the general population by gender

	Men			Women	
		Death by suicide (% of suicides at time of death) *N* = 3,341	Gen. population (% of person-years) *N* = 15,888,346	Death by suicide (% of suicides at time of death) *N* = 1,390	Gen. population (% of person-years) *N* = 15,184,337
**Consultation last year**					
*Mental healthcare*		43.0	4.4	63.2	6.3
*Primary healthcare*		22.0	8.0	20.6	12.6
*No consultation*		34.9	87.6	16.2	80.9
**Consultation last month**					
*Mental healthcare*		23.9		38.6	
*Primary healthcare*		16.7		20.0	
*No consultation*		59.4		41.4	
**Education**					
*High school or less*		81.2	66.3	66.8	53.0
*Higher education*		18.8	33.6	33.2	47.0
**Employment**					
*No employment*		47.8	20.6	60.9	23.4
*Employment*		52.2	79.4	39.1	76.9
**Income**					
*Lowest tertile*		43.7	23.6	54.9	34.1
*Middle tertile*		31.9	30.3	32.7	40.4
*Highest tertile*		24.5	46.1	12.4	25.6

The percentage of suicide deaths by consultation status the last year and month prior to suicide, educational attainment, employment status and income, among men and women, as well as the percentage of person-years in the general population by consultation status the last year, educational attainment, employment status and income, among men and women aged 20–64 years in the period 2009–2021.

Suicides are more common among individuals with lower socioeconomic status (SES). Both men and women who die by suicide tend to have lower education and income, and to have no employment, than the general population. Specifically, 19% of men who die by suicide have higher education, while one-third of men in the general population do. For women, the equivalent shares are one-third for suicides and 47% for the general population. Additionally, a smaller proportion of individuals who die by suicide are employed compared to the general population. Over half of women who die by suicide are non-employed, whereas only one out of four women in the general population are. Lastly, those who die by suicide are underrepresented in the highest income tertile, with nearly half of men and over 50% of women are in the lowest income tertile.

### Consultations for mental health problems last year prior to suicide

#### Educational attainment

**Fig. 1:**
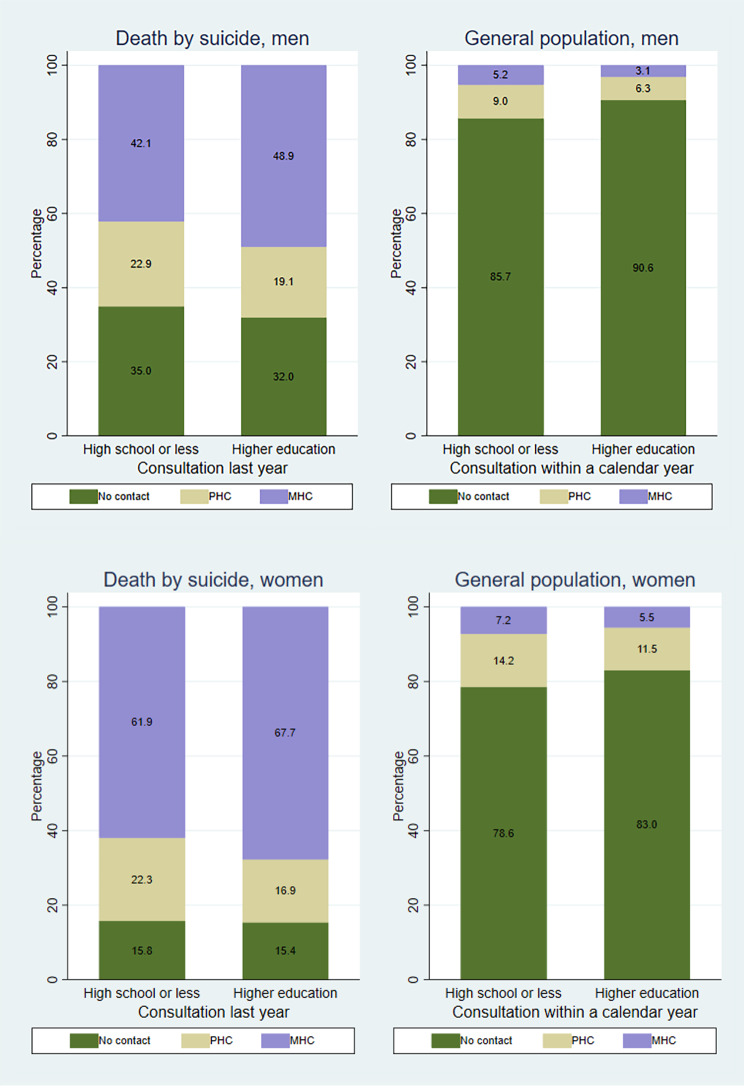
Mental health consultations by educational attainment The distribution of healthcare consultations for mental health problems last year prior to suicide, and percentage of person-years with consultations for mental health problems within a year for the general population, according to educational attainment and gender Higher shares of healthcare consultations for mental health problems last year prior to suicide among higher educated men and women, and lower percentage of person-years with consultations for mental health problems within a year for higher educated men and women in the general population

Fig. 1 shows healthcare usage for mental health problems in the year before suicide and within a calendar year, categorized by educational level. Among men who die by suicide, those with higher education have significantly higher consultation rates with MHC services (49%) compared to those without (42%) (χ2 8.95; df 2 *p* = 0.01). This contrasts with men in the general population, where higher education correlates with lower MHC consultation rates. This is supported by the regression estimates, where we found positive interactions between higher education and suicide for ORs of both PHC and MHC consultations for men (see Table 2 in Supplementary). For women who die by suicide, a similar pattern emerges: 68% of women with higher education consult MHC services in the year before suicide, compared to 62% of those without (χ2 6.58; df 2 *p* = 0.04). Conversely, the latter group has a 22% PHC consultation rate, while those with higher education have 17%. These patterns differ from women in the general population, where higher education is associated with smaller shares of both MHC and PHC consultations. We did, however, only find statistically significant interaction between higher education and suicide on the OR for MHC consultations, not for PHC consultations for women (see Table 2 in Supplementary).

#### Employment status

**Fig. 2:**
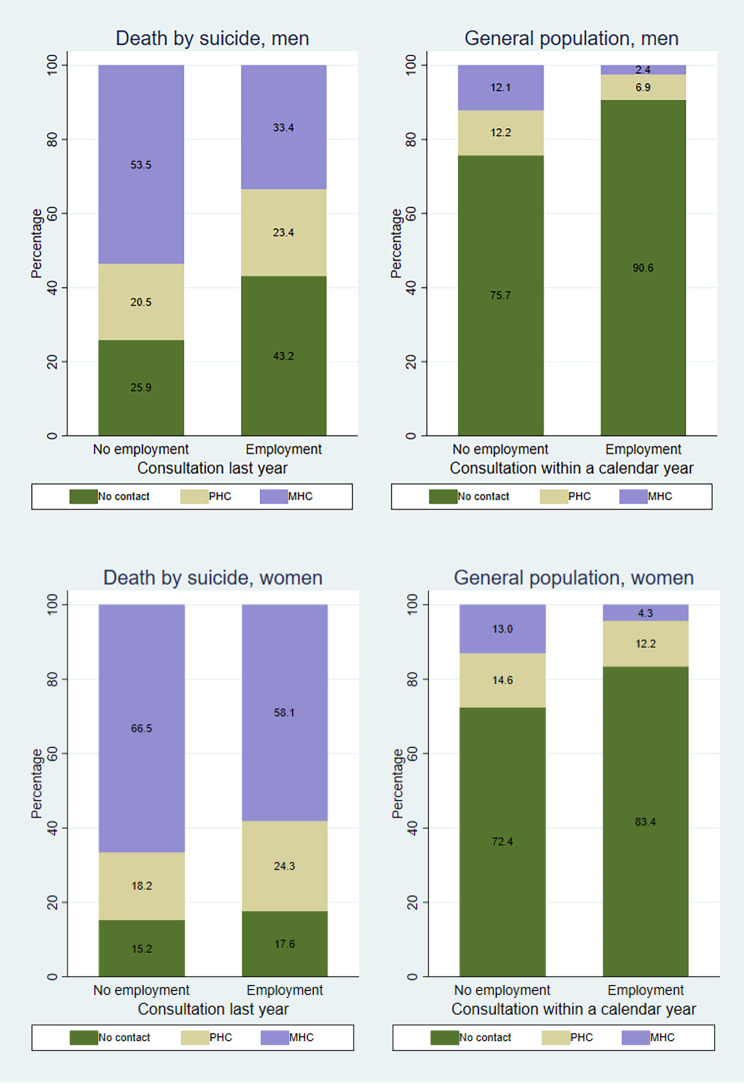
Mental health consultations by employment status The distribution of healthcare consultations for mental health problems last year prior to suicide, and percentage of person-years with consultations for mental health problems within a year for the general population, according to employment status and gender Higher shares of healthcare consultations for mental health problems last year prior to suicide among non-employed men and women, and higher percentage of person-years with consultations for mental health problems within a year for non-employed men and women in the general population

Fig. 2 reveals significant differences in mental health care usage before suicide based on employment status for both men and women (χ2 194.01; df 2 *p* = 0.00 for men and χ2 7.17; df 2 *p* = 0.03 for women). Men who die by suicide and men in the general population exhibit the same pattern, unlike variations by educational attainment. Among those who die by suicide, the disparity is largest in MHC consultations: 54% of the non-employed had a consultation, compared to only one-third of the employed. For men in the general population, the equivalent shares are 12% and 2%. Among women who die by suicide, employed women are slightly less likely to have had a consultation compared to the non-employed. Over half of employed women who die by suicide have had at least one MHC consultation, while non-employed women reach as high as 67%. Interestingly, employed women in the general population have a lower share of MHC consultations but a slightly higher share of PHC consultations compared to the non-employed. Overall, healthcare usage for mental health problems seems more influenced by employment status than educational attainment. The logistic regression models show that being employed substantially decrease the odds of any consultation for mental health problems for both genders (see Table 3 in Supplementary).

#### Income

**Fig. 3:**
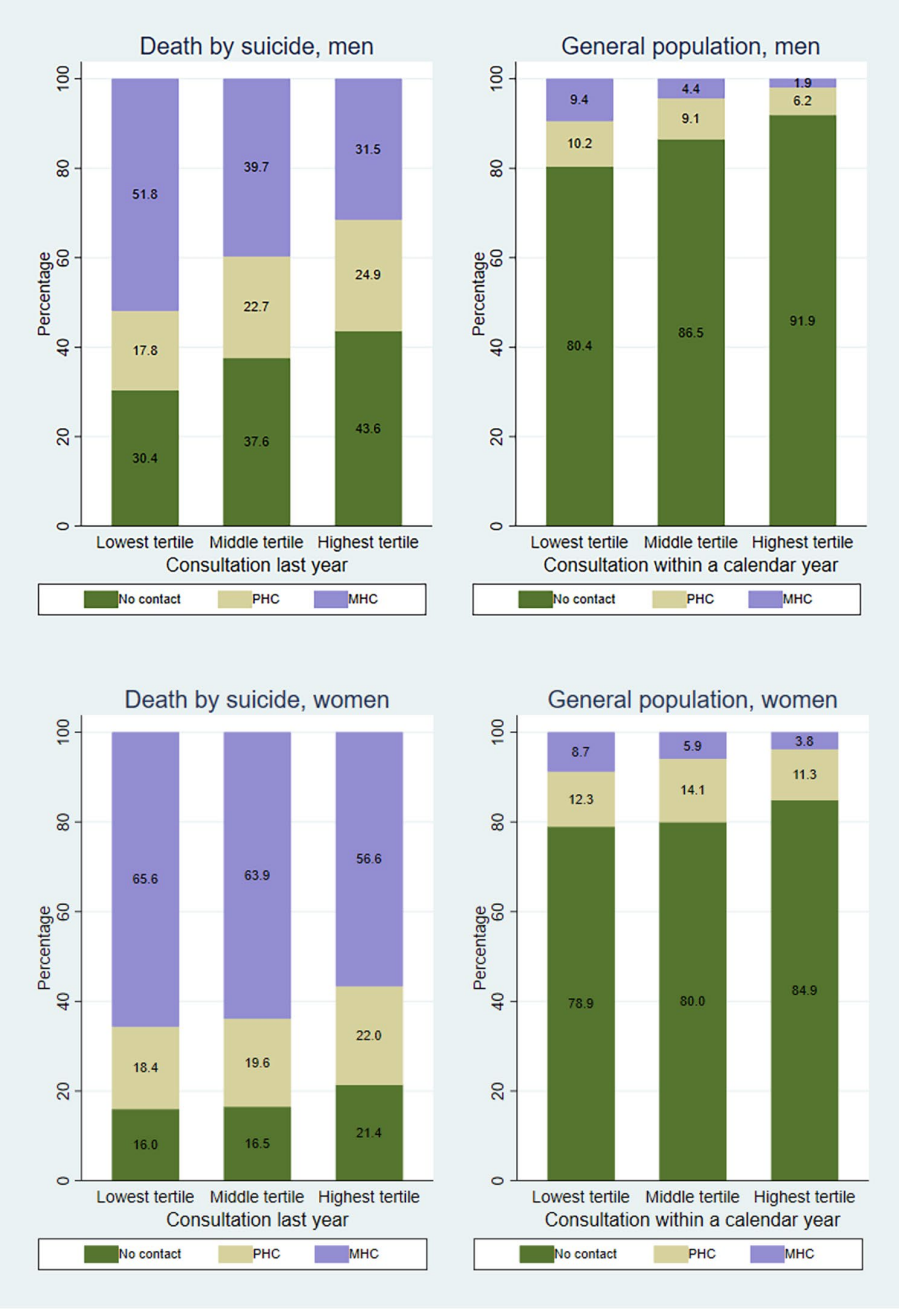
Mental health consultations by income level The distribution of healthcare consultations for mental health problems last year prior to suicide, and percentage of person-years with consultations for mental health problems within a year for the general population, according to income and gender Lower shares of healthcare consultations for mental health problems last year prior to suicide among men and women with higher income, and lower percentage of person-years with consultations for mental health problems within a year for men and women with higher income in the general population

Fig. 3 displays healthcare usage by income distribution. We found significant differences in utilization for mental health problems according to level of income both among men who died by suicide (χ^2^ 92.62; d*f* 4 *p* = 0.00) and among men in the general population. Men in the lowest income tertile have higher shares with MHC consultations and smaller shares of PHC consultations prior to suicide, compared to those in the highest income tertile. This pattern also applies to men in the general population. The estimates from the regression models also indicate a close to linear association for men, for the OR of any mental health consultation, for both those who die by suicide and population in general (see Table 4 in Supplementary). Among women who die by suicide there is no significant difference in mental healthcare utilization according to income distribution (χ^2^ 5.77; d*f* 4 *p* = 0.22). In the general population women in the highest income tertile have both a significantly lower total healthcare usage for mental healthcare problems and smaller share with MHC consultations. The regression models indicate significantly lower OR for any mental health consultation for both those who die by suicide and women in general in the highest income tertile. However, those women in the middle income tertile have the highest OR for a PHC consultation, while women in the lowest income tertile have higher OR of a MHC consultation compared to those with at least a PHC consultation within a year (see Table 4 in Supplementary).

#### Consultations for mental health problems last month prior to suicide

**Fig. 4:**
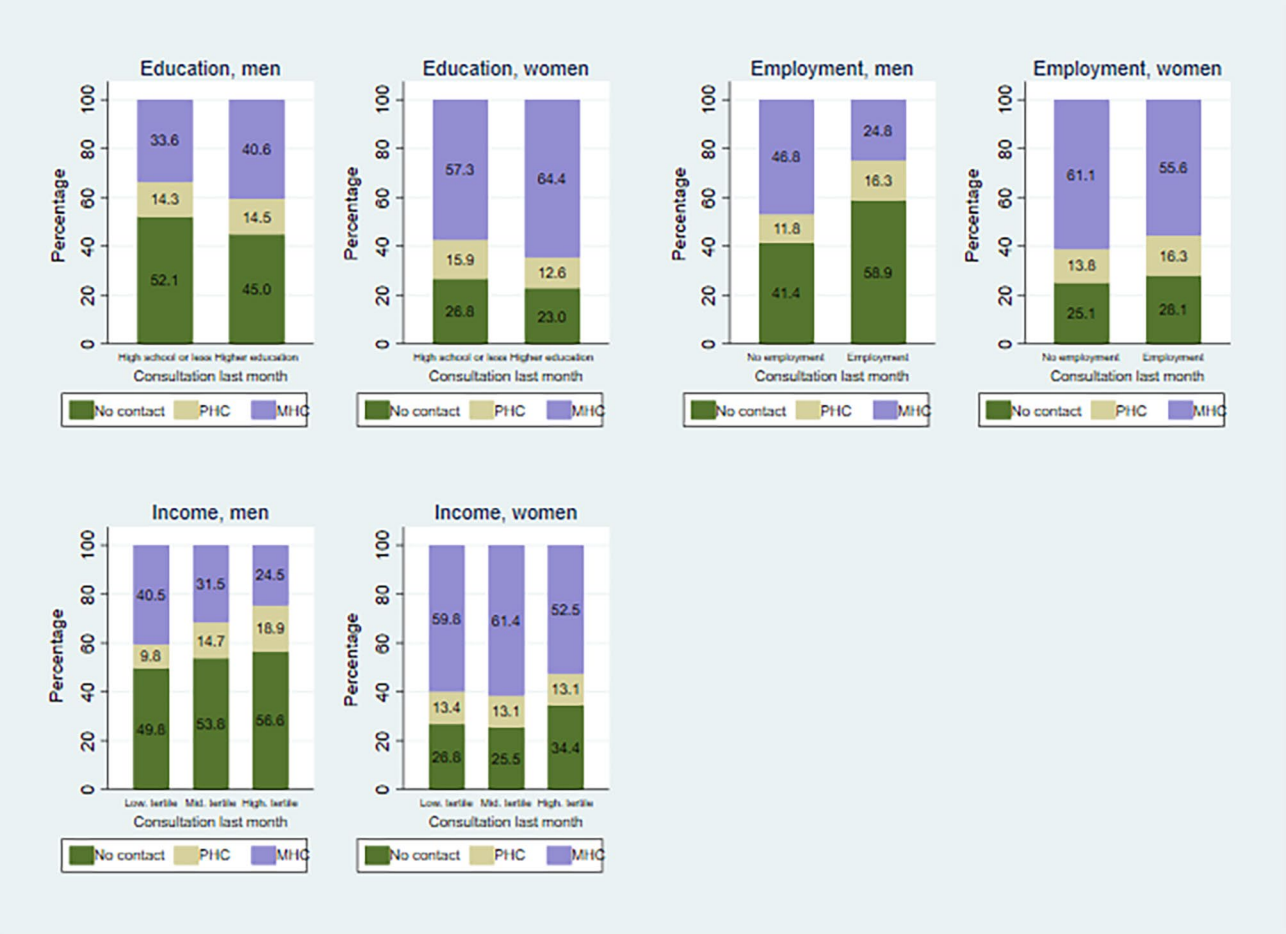
Mental health consultations last month prior to suicide The distribution of healthcare consultations for mental health problems last month prior to suicide, according to educational attainment, employment status and income distribution Lower shares of healthcare consultations for mental health problems last month prior to suicide among men and women without higher education, employed men and women, and men and women with higher income

Fig. 4 presents data on the consultations with PHC or MHC in the month preceding suicide, broken down by measures of SES. The healthcare usage for mental health problems in the month before suicide follows a similar pattern to the usage in the last year, as shown in Fig. 4 and 1. Both men and women with higher education had significantly higher rates of MHC consultations in the last month before suicide compared to those with lower educational levels (χ2 15.59; df 2 *p* = 0.00 for men and χ2 8.79; df 2 *p* = 0.02 for women). Additionally, employed men had lower rates of MHC consultations compared to non-employed men, while the difference in PHC usage between employed and non-employed men was smaller. This is similar to the pattern for mental health care use the last year (see Fig. 2). The difference in mental healthcare utilization between employed and non-employed women is lower the last month than the last year prior to suicide. In fact, whereas there is a significant difference between employed and non-employed men (χ2 56.88; df 2 *p* = 0.00), this is not the case for women (χ2 2.36; df 2 *p* = 0.31).

For men, there is a significant variation in healthcare utilization for mental health problems in the month before suicide, in relation to income distribution (χ2 32.08; df 4 *p* = 0.00). Men in the lowest income tertile had the highest proportions of MHC consultations in the last month before suicide, while men in the highest income tertile had the highest proportions of PHC consultations. For women, there were few disparities in mental healthcare utilization in the month before suicide according to income, similar to the pattern observed in the last year before suicide (χ2 4.62; df 4 *p* = 0.33). The overall mental healthcare utilization in the month before suicide was higher for women compared to men.

## Discussion

Notably, there are disparities in healthcare usage for mental health problems across the different measures of SES. Men and women with higher education who die by suicide exhibit higher overall use, including larger proportions with MHC consultations, compared to those with high school completion or less. These findings are statistically significant and highlight the importance of educational attainment in accessing specialized mental healthcare services. This is also supported by educational level being the only statistically significant aspect of SES the last month prior to suicide for both genders. Health literacy is closely related to educational level, and this finding may thus point to this being a key factor to be referred into specialist healthcare services, as noted by others [17, 29]. The GP can function as a barrier into specialist mental healthcare, and those with higher education may be more equipped with the knowledge as to how to explain the situation [24, 29].

The difference in mental healthcare utilization between employed and non-employed individuals may be more clinically relevant. Employment status exhibits a different aspect of SES, as it can refer to the present level of functioning, rather than embodied knowledge. Those without employment have a higher total utilization and MHC usage than the employed. This could be due to the fact that non-employed individuals, who can be experiencing mental health problems that may form the cause of both suicide risk and non-employment, seek more help from mental healthcare services. Non-employment can also increase the already existing mental health problems. This finding highlights the impact of employment status on mental health and the need for targeted interventions for this population.

The association between income and mental healthcare consultations prior to suicide is slightly more nuanced than that of employment status. Men in the highest income tertile have the lowest overall use and lowest proportions of MHC consultations. This suggests that men in higher income brackets may face barriers to seeking mental healthcare, possibly due to factors such as self-perception, societal expectations, or stigma surrounding mental health. For some men, being overachievers can be a mental coping strategy, and contacting the healthcare services for mental health problems may be in violation with their own self-esteem or masculinity ideal [30]. These men often have no history of mental health problems, and the suicides can seem unexpected. Our study shows that the shares without any consultation for mental health problems among those in the highest income tertile were 43% the last year and 62% the last month prior to suicide. Thus, it is plausible that some of these suicides are perceived as sudden by the bereaved because of the apparent success and the lack of known mental health problems. However, our results cannot state the causes of the suicides. Still, they underline the importance of considering these factors when designing interventions to ensure that individuals in higher income brackets receive the appropriate support and care. This underlines the relevance of this finding.

The associations between SES and healthcare use for mental health problems prior to suicide are more pronounced for men than for women. First, there are larger variations by the different measures of SES among men compared to women. Women follow the same pattern as men regarding educational attainment and employment status, but the differences are smaller, and it is only educational level that is statistically significant the last month prior to suicide. Further, we find a clear relationship between income and likelihood of consultations with mental healthcare services the last year or last month prior to suicide for men, but not for women. The results for women show a much higher healthcare use for mental health problems than men have. We cannot conclude why based on these results, but it may seem that help-seeking for mental health problems is less of a stigma for women, regardless of SES. Women may also to a larger extent recognize symptoms of mental health problems than men. For men, particularly those with high income, healthcare use for mental health problems can be more of a stigma and in violation of the masculinity ideal.

Our results show different associations between mental healthcare use prior to suicide and the components of SES. This is an important finding as it reveals heterogeneity in the high-SES group and shows the need for multiple measurements of SES in studies regarding healthcare use for mental health problems prior to suicide. Otherwise, there is a risk that groups who to a lesser extent under-use healthcare services for mental health problems prior to suicide is overlooked. These results also somewhat contradict previous results, that mostly report low use of MHC for mental health problems in the low-SES groups [13, 16, 18]. We also found low use among the group without higher education, but not among the non-employed or the low-income groups. This discrepancy with previous research may be due to Norwegian-specific factors influencing the results, or due to the inclusion of several measurements of SES.

### Implications

The results can indicate that men without higher education, and men with employment and middle- and high-income under-use the mental healthcare services, as 36–44% of men in these groups have no contact with healthcare services for mental health problems prior to suicide. Here there might be an unused potential for suicide prevention through measures for increased mental healthcare use. Means for increasing health literacy in these groups can be helpful for communicating mental health problems to the GP or others. Measures targeting men with the emphasis on coping with stressful life events should be explored, as well as striving to reduce mental health problems as a stigma that violates the masculinity ideal.

There are multiple studies that states an association between non-employment and/or low income and increased risk of suicide. These groups, however, seem to utilize the healthcare services for mental health problems to a higher degree than the employed and/or those with high income. It is somewhat expected that the non-employed and those in the lowest income tertile have high levels of MHC service use because this group might have their income fully or partly based on welfare benefits. In order to obtain such benefits from the Norwegian Labour and Welfare Administration a doctor’s certificate is required. Increased mental healthcare usage is perhaps not the most fruitful suicide prevention methods in these groups. For these groups the solution to suicide prevention may lie outside of the healthcare system. A more effective strategy could be increasing employment rate and/or income and welfare benefits in the entire population. This is in line with a systematic review stating that prevention strategies focusing on lower socioeconomic strata can be as effective as measures targeting psychiatric risk factors, at the populational level [31].

### Strengths and limitations

The main advantage of this study is that the dataset provides information regarding almost all consultations for mental health problems prior to suicide. To our knowledge this is the first population-wide study that includes both PHC and MHC consultations and that investigates patterns of service use by SES. By including multiple measures of SES, the results shed light on different associations between healthcare use for mental health problems prior to suicide and the components of SES. The use of register data also minimizes the problem of selection bias as it includes all the suicide deaths in the period 2009 to 2021 in the age group 20 to 64.

The study also has some limitations. Firstly, we do not have information on somatic consultations prior to suicide. This means that some of the individuals in the no consultations groups may have had somatic consultations instead. Our dataset lacks information regarding the use of private healthcare providers, such as private psychologists. The increasing use of private healthcare providers limits the gatekeeping role of the GP, particularly for the high-income group. Although the overall use of private psychologists is limited at the population level due to expenses, it may contribute to underestimate the shares with MHC consultations. Further, two of the measures of SES are binary, giving simplified versions of both educational level and employment status. For the latter, using a binary variable that incorporates everyone with at least one hour per week as employed results in inclusion of some with very low number of work hours per week. These individuals may not experience the full positive financial or social effects of employment. It is also a limitation that employment measured the year prior to the year of death because it reduces the possibility to consider changes in employment status close to the suicide. Also, the analyses in this study cannot be used to establish any causal effects. In the current study we have utilized data on the entire Norwegian population, but with a matching-approach it may be possible to isolate the impact of the SES-factors on healthcare use for mental health problems prior to suicide to a greater extent, but such an approach would also increase the risk of selection bias in comparison to the selected approach [32].

### Future research

Our results show that men and women with higher education who die by suicide have higher use of MHC services, but this is not enough to state that those in the lower SES group in Norway have less access to MHC services. In fact, the measures of SES in the current study have opposite associations. Future research should investigate the factors of SES in combination. The current study is highly descriptive, and the aim is to form the basis for further and more advanced analyses. By utilizing more sophisticated statistical methods future research may come one step closer to establishing any possible causal relationships between SES and mental healthcare use. Survival analysis could also be utilized in order to investigate differences between the different SES-groups and/or the groups using the different types of healthcare.

The results from the current study uncover several groups that have no consultations with either PHC or MHC services for mental health problems prior to suicide. Future research should investigate these groups in more detail, both regarding potential somatic consultations and stressful life events. By identifying possible common traits, it is attainable to build a knowledge base for further suicide prevention measures in a group that is treated primarily outside the MHC services.

The current study includes all Norwegian residents, but we have not divided by immigration status. Future studies should investigate the healthcare usage for mental health problems prior to suicide by SES in the immigrant population, to see if the associations in these groups are different from the results in the current study. Studies have shown that foreign born immigrants and Norwegian born with two foreign born parents have lower mental healthcare utilization than the majority population, both in general and prior to suicide [9, 33]. The association between healthcare use for mental health problems and SES prior to suicide may also be different for the immigrant population than for the majority.

## Conclusion

The results presented here show that there are differences in healthcare use for mental health problems prior to suicide by SES. However, there are differences in associations by the three different components of SES; educational attainment, employment status and income distribution. This is important because it unmasks the heterogeneity of both the high and low SES groups. Educational level has a different association with mental health consultations for those who die by suicide and the general population, as opposed to the other components. The associations are also gendered. While some of the groups with higher suicide risk, like the non-employed and those with low income, have relatively high mental healthcare utilization prior to suicide, the findings are opposite for educational attainment. Employed men with high income and high school completion or less appear to have low level of consultations for mental health problems prior to suicide and targeted measures for increasing this should be explored. At-risk groups, like the non-employed and/or those with low income, on the other hand, utilize healthcare services for mental health problems to a high extent prior to suicide. The same is true for women, where the healthcare utilization for mental health problems prior to suicide is high for all groups, independent of SES-category or timespan. This means that most of the women who die by suicide are known to the healthcare professionals. Improved methods for recognizing at-risk individuals should be investigated.

## Electronic supplementary material

Below is the link to the electronic supplementary material.

Supplementary Material 1

## Data Availability

The data that support the findings of this study are available from the Cause of Death Registry, the Norwegian Patient Registry, the Control and Payment of Health Reimbursements Database, the Social Security Database and Statistics Norway, but restrictions apply to the availability of these data, which were used under license for the current study, and so are not publicly available. Data are however available from the authors upon reasonable request and with permission of the Cause of Death Registry, the Norwegian Patient Registry, the Control and Payment of Health Reimbursements Database, the Social Security Database and Statistics Norway.

## Abbreviations

SES: Socioeconomic status
GP: General practitioner
PHC: Primary healthcare
MHC: Mental healthcare
SHC: Specialist healthcare
OECD: Organization for Economic Cooperation and Development
CDR: Cause of Death Registry
NPR: Norwegian Patient Registry
KUHR: Control and Payment of Health Reimbursements Database
DSF: Norwegian Population Registry
FD-trygd: –Social security database
NAV: Norwegian Labour and Welfare Administration
ICD-10: –International Classification of Diseases
ICPC-2: –International Classification of Primary Care
BUP: Children and adolescents’ psychiatric services
VOP: Adult mental healthcare
TSB: Specialized substance use treatment
