# Supplementary material for: Variations in healthcare utilization for mental health problems prior to suicide by socioeconomic status: a Norwegian register-based population study
Source: BMC Health Serv Res. 2024 May 21;24:648. doi: 10.1186/s12913-024-11113-w (PMC11110240; doi:10.1186/s12913-024-11113-w)
Supplement: Supplementary file 1 — Supplementary Material 1 [file 12913_2024_11113_MOESM1_ESM.docx]

Table 2: Odds ratio for at least one PHC consultation for mental health problems compared to those without any mental health consultations within a year, by gender.

| PHC vs none | Men (N=14,619,127) | | Women (N=13,850,219) | |
| --- | --- | --- | --- | --- |
|  | OR | CI | OR | CI |
| **Suicide** | 4.90*** | 4.39 – 5.47 | 6.86*** | 5.49 – 8.57 |
| **Suicide*higher education** | 1.60** | 1.22 – 2.09 | 0.79 | 0.52 – 1.20 |
| **Higher education (base=high school or less)** | 0.76*** | 0.76 – 0.76 | 0.83*** | 0.82 – 0.83 |
| **Employment (base=no employment)** | 0.54*** | 0.54 – 0.54 | 0.68*** | 0.68 – 0.68 |
| **Income (base=middle tertile)** |  |  |  |  |
| *Lowest income tertile* | 1.03*** | 1.03 – 1.04 | 0.81*** | 0.80 – 0.80 |
| *Highest income tertile* | 0.69*** | 0.69 – 0.69 | 0.80*** | 0.80 – 0.81 |
| **Age group (base=35-49)** |  |  |  |  |
| *20-34* | 0.79*** | 0.79 – 0.80 | 0.81*** | 0.80 – 0.81 |
| *50-64* | 0.94*** | 0.93 – 0.94 | 0.94*** | 0.93 – 0.94 |
| _cons | 0.20*** | 0.20 – 0.21 | 0.29*** | 0.29 – 0.29 |
| Log likelihood | -4154590.4 | | -5516664.2 | |

*p < .05; **p < .01; ***p < .001

Table 3: Odds ratio for at least one MHC consultation for mental health problems compared to those without any mental health consultations within a year, by gender.

| MHC vs none | Men (N=14,067,741) | | Women (N=12,885,795) | |
| --- | --- | --- | --- | --- |
|  | OR | CI | OR | CI |
| **Suicide** | 14.83*** | 13.40 – 16.40 | 32.89*** | 27.03 – 39.40 |
| **Suicide*higher education** | 2.77*** | 2.19 – 3.49 | 1.71** | 1.22 – 2.40 |
| **Higher education (base=high school or less)** | 0.76*** | 0.75 – 0.76 | 0.91*** | 0.90 – 0.91 |
| **Employment (base=no employment)** | 0.23*** | 0.23 – 0.23 | 0.28*** | 0.28 – 0.28 |
| **Income (base=middle tertile)** |  |  |  |  |
| *Lowest income tertile* | 1.37*** | 1.36 – 1.38 | 0.91*** | 0.91 – 0.92 |
| *Highest income tertile* | 0.52*** | 0.51 – 0.52 | 0.72*** | 0.71 – 0.72 |
| **Age group (base=35-49)** |  |  |  |  |
| *20-34* | 0.86*** | 0.86 – 0.87 | 1.06*** | 1.05 – 1.06 |
| *50-64* | 0.63*** | 0.62 – 0.63 | 0.63*** | 0.62 – 0.63 |
| _cons | 0.19*** | 0.19 – 0.19 | 0.24*** | 0.23 – 0.24 |
| Log likelihood | -2446343.3 | | -3195994.1 | |

*p < .05; **p < .01; ***p < .001

Table 4: Odds ratio for at least one MHC consultation for mental health problems compared to those with at least one PHC consultation for mental health within a year, by gender.

| MHC vs PHC | Men (N=1,935,172) | | Women (N=2,862,726) | |
| --- | --- | --- | --- | --- |
|  | OR | CI | OR | CI |
| **Suicide** | 3.17*** | 2.84 – 3.54 | 4.88*** | 4.11 – 5.80 |
| **Suicide*higher education** | 1.65*** | 1.28 – 2.14 | 2.02*** | 1.44 – 2.85 |
| **Higher education (base=high school or less)** | 1.18*** | 1.17 – 1.19 | 1.29*** | 1.29 – 1.29 |
| **Employment (base=no employment)** | 0.42*** | 0.42 – 0.43 | 0.39*** | 0.39 – 0.39 |
| **Income (base=middle tertile)** |  |  |  |  |
| *Lowest income tertile* | 1.34*** | 1.33 – 1.35 | 1.18*** | 1.17 – 1.19 |
| *Highest income tertile* | 0.76*** | 0.76 – 0.77 | 0.89*** | 0.89 – 0.90 |
| **Age group (base=35-49)** |  |  |  |  |
| *20-34* | 1.14*** | 1.13 – 1.15 | 1.39*** | 1.38 – 1.39 |
| *50-64* | 0.68*** | 0.68 – 0.69 | 0.66*** | 0.66 – 0.66 |
| _cons | 0.87*** | 0.87 – 0.88 | 0.77*** | 0.77 – 0.78 |
| Log likelihood | -1184739.5 | | -1725534.3 | |

*p < .05; **p < .01; ***p < .001

Figure title: Figure 5: Mental health consultations by educational attainment.


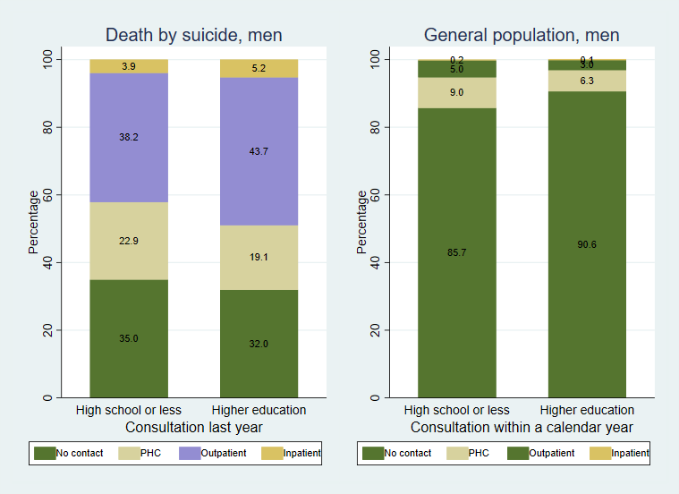

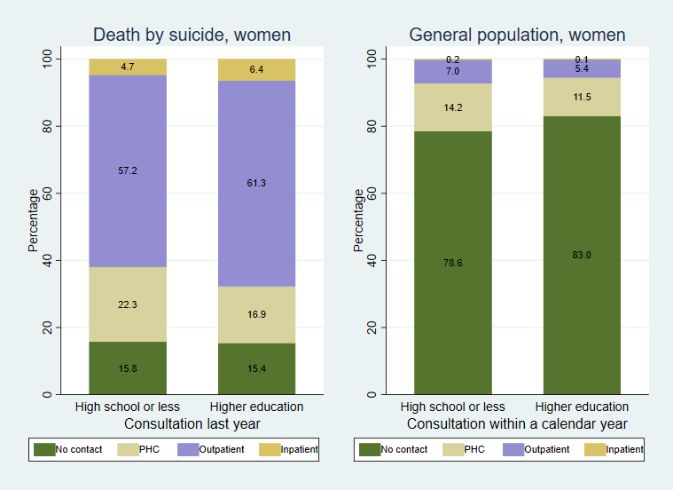


Legend: Figure 5: The distribution of healthcare consultations for mental health problems last year prior to suicide, and percentage of person-years with consultations for mental health problems within a year for the general population, according to educational attainment and gender.

Figure title: Figure 6: Mental health consultations by employment status.


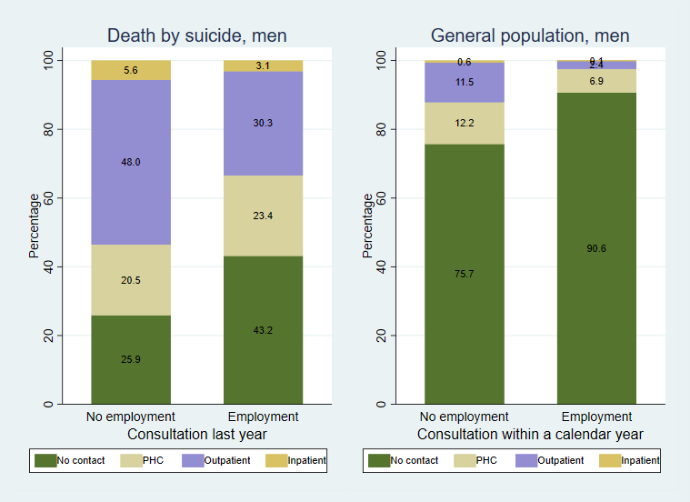

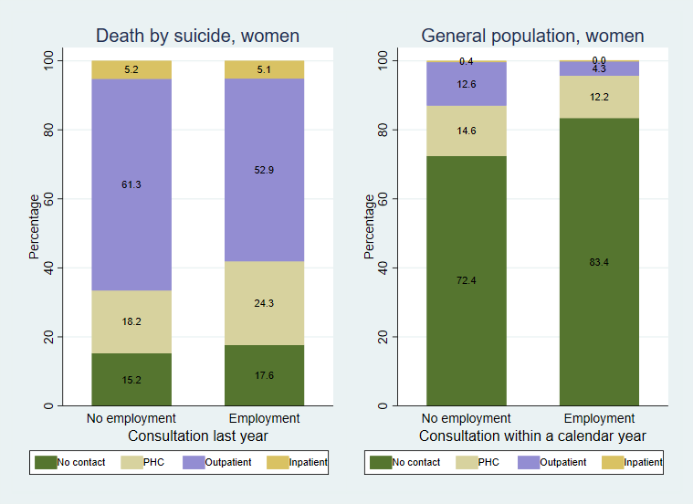


Legend: Figure 6: The distribution of healthcare consultations for mental health problems last year prior to suicide, and percentage of person-years with consultations for mental health problems within a year for the general population, according to employment status and gender.

Figure title: Figure 7: Mental health consultations by income level.


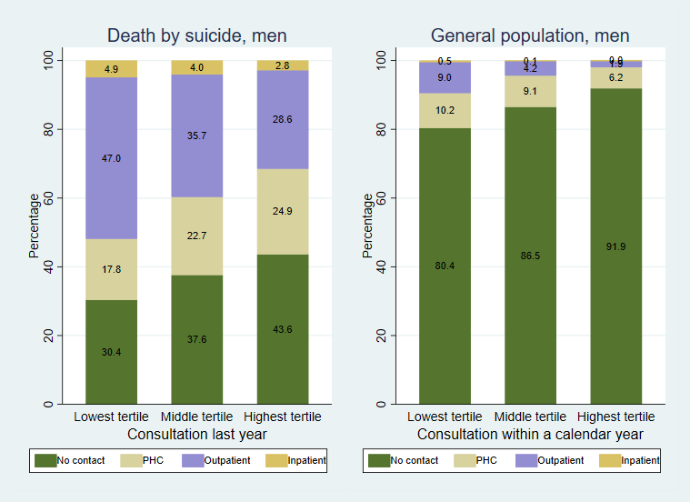

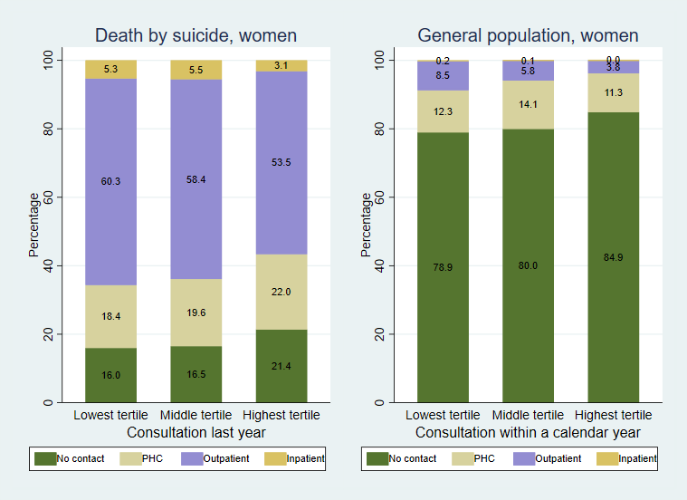


Legend: Figure 7: The distribution of healthcare consultations for mental health problems last year prior to suicide, and percentage of person-years with consultations for mental health problems within a year for the general population, according to income and gender.
